# Design Training and Creativity: Students Develop Stronger Divergent but Not Convergent Thinking

**DOI:** 10.3389/fpsyg.2021.695002

**Published:** 2021-10-04

**Authors:** Tiansheng Xia, Mengxia Kang, Meng Chen, Jia Ouyang, Fei Hu

**Affiliations:** School of Art and Design, Guangdong University of Technology, Guangzhou, China

**Keywords:** design training, creativity, divergent thinking, convergent thinking, cognitive flexibility

## Abstract

Design training programs that teach creativity often emphasize divergent thinking (generation of ideas) more than convergent thinking (evaluation of ideas). We hypothesized that training would lead to more both types of creativity, but especially divergent thinking. Three groups of university students (*N*=120; *n*=40 in each group) were recruited to participate: senior design students (graduate students with at least 4years of design training as undergraduates); junior design students (undergraduates in their first year of design training); and undergraduate students in majors unrelated to design. The students completed three tasks in a classroom setting to assess divergent thinking (Alternate Uses Task), convergent thinking (Remote Associates Task), and nonverbal abstract reasoning (Raven’s Progressive Matrices Test). The results of one-way ANOVAs showed that as expected, senior design students significantly outperformed junior design students and non-design majors in divergent thinking. However, contrary to expectations, senior design students had significantly lower scores than the non-design group on convergent thinking; the junior design students’ scores fell in the middle but were not significantly different from either of the other groups. There were no group differences in nonverbal abstract reasoning. These findings suggest that design training significantly improves students’ ability to generate ideas but does not improve, or may even hinder, their ability to evaluate whether the ideas are useful for the task at hand. The results have implications for developing a research-based curriculum in design training programs.

## Introduction

Industrial designers are commonly assumed to be more creative than other people, because design requires creativity (Sarkar and Chakrabarti, 2011). Design ability is often applied to developing new products (Er Biyikli and Gulen, 2018; Lazar, 2018), which requires the innovative use of variables in the environment (physical objects, behaviors, rules, etc.) to result in favorable outcomes for the executor. There is a close relationship between creativity and design, and for a long time, creativity has been regarded as an important criterion in the evaluation of designers’ proficiency (Sundström and Zika-Viktorsson, 2003). Therefore, many design programs have set up courses to improve students’ creativity (Cheung et al., 2003, 2006; Wang, 2008). However, instructors in most training programs conceptualize creativity as a whole without examining its component parts, or they emphasize some components and not others (Sarkar and Chakrabarti, 2011). Research on this topic can help in developing a design curriculum that covers all areas of creativity.

As early as 1950, Guilford conceptualized creativity as a combination of two forms of thinking, namely, divergent thinking and convergent thinking (Guilford, 1950). Divergent thinking broadens the representational research space while convergent thinking is used to identify the best ideas for the task at hand (Cortes et al., 2019). These dual processes influence the overall process of creation (Barr, 2018), although there may be times when one form of creativity is more influential than the other (Sarkar and Chakrabarti, 2011; Lubart, 2016; Webb et al., 2017; Zhang et al., 2020). The designer can switch between the two forms of thinking according to the actual task requirements (Lazar, 2018). The alternating pattern of divergent thinking and convergent thinking forms the creative process (de Vries and Lubart, 2017).

The creative process can be divided into two stages, with divergent thinking being prominent early in the process and convergent thinking being prominent later in the process. First, in the idea generation stage, the designer uses mostly divergent thinking to put forward as many abstract ideas, forms, and design schemes as possible (Forthmann et al., 2019). During this first stage, distraction is beneficial and creative generation may depend on the availability of unfiltered, low-level perceptual information (Weinberger et al., 2017). Second, in the idea evaluation stage, the designer uses mostly convergent thinking to evaluate these ideas and to determine a solution, resulting in an answer that is not just novel but also useful for the purposes at hand (Guilford, 1957). In this stage, concentration is needed to evaluate the rationality and feasibility of the design scheme (Mohamed, 2016). This stage requires task-directed thoughts and the integration of semantically distant concepts (Weinberger et al., 2017).

Although both divergent and convergent thinking are thought to be important for creativity, there are two reasons to expect that design training might help in developing divergent thinking more than convergent thinking. The first reason is that training programs give more attention to divergent thinking (Rao et al., 2021). Some have asserted that a cognitive process can be judged as creative only if it causes divergent thinking (Finke et al., 1992). Therefore, some early evaluations of creativity focused on the novelty, fluency, and flexibility of ideas (Haritaipan et al., 2018).

The results of two recent studies on the creativity of first-year and senior engineering students suggest that the training emphasis on one thinking may come at the expense of improvements in another thinking. First-year students scored significantly higher on the design thinking scale, while senior students performed significantly better on the integrative thinking scale (Coleman et al., 2020). First-year students also generated significantly more solutions than seniors and showed higher activation in the brain region associated with cognitive flexibility and divergent thinking (Hu et al., 2021).

Design students may perform better on tasks of divergent than convergent thinking because divergent thinking can be taught even in a short period, but there is no corresponding evidence that convergent thinking can be taught. Tran et al. (2020) conducted a 14-week undergraduate creative methods course and found that the participants demonstrated significant promotion in divergent thinking at the post-test. Similarly, Rao et al. (2021) found that training in design thinking significantly increased ideational fluency and elaboration in a divergent thinking task. Another study documented that analogies training improved design consultants’ innovations and divergent thinking (Kalogerakis et al., 2010). Finally, training in cognitive flexibility, which is correlated with divergent thinking (Benedek et al., 2012; Zabelina et al., 2012), has been shown to have direct and near transfer effects (van Bers et al., 2020).

The second reason to expect senior design students to show better divergent than convergent thinking is that the use of divergent thinking (encouraged in training programs) may inhibit convergent thinking. Research in cognitive psychology has shown that a person’s cognitive style is mainly characterized by either divergent or convergent thinking, suggesting that one approach to thinking may hinder the other approach (Kuypers et al., 2016). The implication for design training is that the enhancement of divergent thinking may inhibit the development of convergent thinking (Yue and Gong, 1999; Hommel et al., 2011; Kuypers et al., 2016).

The aim of the current research was to address this question: What is the specific impact of design training on design majors’ convergent and divergent thinking? Our general assumption was that design training would improve both types of creativity, but this would be especially evident in their divergent thinking. To test this assumption, we compared three groups of university students: senior design students (graduate students who already had at least 4years of design training); junior design students (undergraduates in the first year of a design program); and students who were not majoring in design. The three groups were compared on tests of divergent thinking, convergent thinking, and nonverbal abstract reasoning.

Since divergent thinking might benefit from a minimum cognitive-control state and so that the individual can easily “jump” from one thought to the other. However, convergent thinking is likely to benefit from strong top-down cognitive-control state and so that the individual can quickly conduct subsequent performance in tasks. The training of one thinking may impair the performance of the other (Hommel et al., 2011). The specific hypotheses were (1) senior design students will perform better on divergent thinking tasks rather than convergent thinking tasks, when compared with junior design students and non-specific majors; (2) the difference in creativity between senior design students and the other two groups of students will be greater for divergent thinking than convergent thinking.

## Materials and Methods

### Participants

Undergraduate and graduate students (*N=* 120; 61 males; mean age=21.2years, SD=2.5) at Guangdong University of Technology participated in this study. They included three groups: senior design students (*n*=40; graduate students majoring in industrial design, visual design, and interaction design with at least 4years of design training); junior design students (*n*=40; first-year undergraduate students majoring in industrial design who did not receive systematic design training in their pre-university studies); and non-design majors (*n*=40; undergraduate or graduate students majoring in management, applied mathematics, or economics, with no design training). The three groups were similar in the proportion of male and female students, and in nonverbal abstract reasoning [measured by Raven’s Standard Progressive Matrices (SPM)], see Table 1. The participants provided written informed consent and received a small payment (CNY 15) after the study. The study protocol was approved by the Ethics Committee of the first author’s institution and was conducted according to the ethical standards established in the 1964 Declaration of Helsinki and its later amendments.

**Table 1:** Number of males and females in the senior design, junior design, and non-design groups, and descriptive information about the study variables in each group.

Sample	Senior design	Junior design	Non-design
*N* (F:M)	22:18	19:21	20:20
RPM	54.45 (5.48)	56.03 (4.16)	55.55 (2.54)
AUT^***^	0.71 (0.96)	−0.40 (0.89)	−0.32 (0.74)
RAT^*^	37.20 (6.00)	38.78 (5.81)	40.40 (6.16)

*N=120. RPM, Raven’s Progressive Matrices; AUT, Alternate Uses Task; and RAT, Remote Associates Task*.

*p<0.05;

*** *p<0.001*.

### Procedure and Design

The study consisted of one 70-min session with one break. The participants signed the written consent form when they arrived. The Alternative Uses Task (AUT; 10min), Remote Associates Test (RAT; 20min), and SPM (40min) were then administered. The participants then received a small payment to thank them for their time and effort.

### Measures

#### AUT (Divergent Thinking)

The AUT (Guilford, 1967) is a test of divergent thinking. Participants are asked to think of, and then write down, as many possible uses as they can for a simple object, such as a brick, shoe, or newspaper. Participants could describe each use as briefly or extensively as they wanted, and the task was terminated after 10min. The task was administered in Chinese for the purposes of this study. Two graduate students independently evaluated each response on four dimensions: originality, fluency, flexibility, and elaboration. The score for originality was given based on the frequency with which the use appeared in the set of uses generated by the full sample. A use that was the same as only 5% of all uses generated by the sample was scored as unusual (1 point) and a use that was the same as only 1% was scored as unique (2 points); otherwise, the score was 0. The fluency score was the number of uses generated. The flexibility score was the number of different categories represented by the items on the list. Elaboration was assessed based on the amount of detail in the list. The scores on the four dimensions were summed to create a total score, and the total scores were standardized.

#### RAT (Convergent Thinking)

A modified Chinese version of the RAT (Mednick, 1962) was designed for the purposes of this study to measure convergent thinking. In the original English language version of the RAT (Mednick, 1962), participants are given three unrelated words (e.g., shelf, worm, and end) and asked to find another word that would form a compound word with each of the three unrelated words (e.g., adding the word “book” could create the compound words “bookshelf,” “bookworm,” and “bookend”). In English, the solution would produce three new words that are not related in meaning. The original word and the three solutions differ in both morphology and in semantics.

Because of the stark differences between the English and Chinese languages in morphology and semantics, the RAT in these two languages is analogous but not parallel tests of convergent thinking. In Chinese, adding the same character to three unrelated words generates new words that differ from each other in morphology (and in the pronunciation of the added character) but are related semantically. Presented with the characters “昼,” “深,” and “晚” (day, deep, and evening), the participant could add the character “夜” (night) to generate “昼夜,” “深夜,” and “夜晚” (day and night, late at night, and night). Depending on the word, the added character would appear either to the right or to the left of the original character and would likely be pronounced differently in the three solutions, but because the character for “night” appears in each new word, the three new words will be related semantically. That is, the three new words would differ from the original word and from each other in morphology, but the three new words and the original word would have shared meaning. There was a 20-min limit to complete the 58 items.

#### SPM (Nonverbal Abstract Reasoning)

Raven’s Progressive Matrices Test is a widely used nonverbal assessment of fluid intelligence, including nonverbal abstract reasoning (Raven et al., 2003). The task measures the individual’s ability to identify perceptual relations and to reason by nonverbal analogy. Sets of cards with drawings and symbols are presented and the examinee is asked to identify patterns within each set of cards. The SPM comprises 60 items, each scored as correct or incorrect. The final score is the number of correct responses. The test takes 40min to administer.

### Statistical Analysis

We performed a mixed-design 3×2 ANOVA with the between subjects factor of group (three groups: senior design students, junior design students, and non-design majors) and within subjects factors of creative tasks (AUT task and RAT tasks), and variables, such as intelligence (RPM), age, and gender, can be treated as covariance in our data analysis.

## Results

The ANOVA revealed a significant main effect of interaction of group×creative task, *F* (2, 114)=8.47, *p*<0.001, $\eta_{p}^{2}$=0.129, indicating that the participants’ performances on the AUT and RAT tasks varied according to group, see Table 1. The interactions of creative task×intelligence, creative task×age, and creative task×gender are not significant, *p_s_ >* 0.05, indicating that these factors have no effect.

Two one-way ANOVAs were performed to test differences across the three groups (senior design students, junior design students, and non-design majors). On the AUT task, there was a significant difference across the three groups, *F* (2, 117)=20.40, *p*<0.001, $\eta_{p}^{2}$=0.259, see Table 1. As expected, pairwise comparisons showed that the senior design students obtained significantly higher scores than junior design students and non-design students, *p_s_*<0.001. There was no significant difference between the junior design students and non-design students, *p*=0.695.

On the RAT task, there was a significant difference across the three groups, *F* (2, 117)=3.14, *p*=0.047, $\eta_{p}^{2}$=0.051, see Table 1. Pairwise comparisons revealed an unexpected pattern of results. The senior design students obtained significantly lower scores than the non-design students, *p*=0.014. There was no significant difference between the junior design students and the non-design students, *p*=0.199, or between the senior design students and the junior design students, *p*=0.227.

In addition, correlation analysis showed that the correlations between gender and other variables were not significant. There was a significant positive correlation between age and AUT scores and a significant negative correlation between age and RPM scores. The correlation between RAT and RPM scores was significant and positive. The correlations between other variables were not significant, see Table 2. We further conducted regression on age within the groups, and all results were nonsignificant.

**Table 2:** Correlations among the study variables.

	1	2	3	4	5
Gender	–				
Age	−0.08	–			
AUT	0.14	0.45^**^	–		
RAT	0.05	−0.09	0.03	–	
RPM	0.05	−0.21^*^	0.07	0.29^**^	–

* *p<0.05;*

** *p<0.01*.

## Discussion

The results of the current study suggest that training in design improves divergent thinking but does not improve and may even lower convergent thinking. Students who were enrolled in a design program for several years had higher divergent thinking scores compared to students who were just beginning a design program and students who had no training in design. These findings are consistent with previous research on training received in typical design programs (Fink et al., 2006; Sun et al., 2016). They are also consistent with the results of an experiment in which, compared to controls, participants who received 20 sessions of training in divergent thinking showed greater changes in neural activity in brain areas linked to this form of creativity (Sun et al., 2016). However, contrary to our hypotheses, the non-design majors had significantly higher convergent thinking than the senior design students.

Many believe that high divergent thinking represents high creativity (Finke et al., 1992; Goldschmidt, 2016) and it may be for this reason that educators who want to increase creativity tend to focus on increasing divergent thinking. In curriculum training of design, the educators emphasize the cultivation of divergent thinking and an open environment where students are encouraged to share their ideas, such as the teaching methods, instructional procedures, and teacher–student relationships (Wang et al., 2010; Wu et al., 2019). These design training is likely to be conducive to divergent thinking.

However, our evidence suggests that the focus on divergent thinking may come at the expense of convergent thinking. First, training in divergent thinking might take time away from training in convergent thinking, resulting in lower scores on convergent thinking tasks. Second, even in training programs that do teach convergent thinking, an increase in divergent thinking may inhibit the development of convergent thinking. The results of this study were consistent with these possibilities, in that senior design students had significantly lower convergent thinking scores than the non-design students. This may have been due to the non-design students having developed greater convergent thinking through training in their majors, but there was no significant difference in the level of convergent thinking between the junior design students and the non-design students, suggesting that having more design training (more than 4years vs. none) did not improve convergent thinking, and may even have harmed it.

Interestingly, some research suggests that the ability to engage in divergent thinking relies in part on a certain level of convergent thinking (Webb et al., 2017; Zhu et al., 2019). Divergent thinking mostly helps in the early stage of design, but convergent thinking is needed to evaluate and hone these ideas in the late stage. Convergent thinking is necessary, and its criterion and skillful use are one key to creativity. The results suggest that students could benefit from design training that fosters both types of thinking without compromising one or the other. This possibility deserves the attention of researchers in education and psychology.

Cognitive flexibility is important to problem solving and is related to creativity. Martindale (2007) conceptualized this individual difference in terms of cognitive inhibition: Highly creative people can flexibly shift their attentional focus when faced with different task requirements—that is, they can inhibit or disinhibit cognition depending on the type of creativity that is needed. In the early stages of the creative process, in which the goal is to produce as many design schemes as possible, the creator is more likely to defocus, and disinhibition of cognition helps divergent thinking. However, in the late stage of the creative process, divergent thinking is less helpful because it leads to slower information processing, reducing the ability to evaluate and integrate design schemes using convergent thinking. Martindale’s model has been supported by empirical research (Cheng et al., 2016). Creators need to suppress irrelevant information to enhance focus during convergent thinking, but can flexibly switch to divergent thinking according to task requirements (Zabelina and Robinson, 2010; Zabelina et al., 2012). This model is consistent with neuroimaging research showing that creative achievements are associated with over-activation of the prefrontal cortex, suggesting that cognitive flexibility promotes more creative ideas (Colombo et al., 2015).

Several limitations to this study are worth mentioning. First, the cross-sectional design does not provide information about changes over time and does not allow conclusions about causality (e.g., Coleman et al., 2020; Hu et al., 2021). A longitudinal design would be helpful in identifying which junior design students went on to complete the design program. Second, we adopted the standard RPM test as the tool to measure nonverbal abstract reasoning, and this measure appeared to be too easy for the college students in our sample. The average scores were in the top 90% based on the measure’s norms, implying a possible ceiling effect for university students. Though Hommel et al.’s (2011) study also used the standard RPM test and obtained similar results, future research using the advanced RPM is likely to obtain more relevant results concerning nonverbal abstract reasoning. Third, the number of students in each group may have been too small to detect significant group differences.

Despite the limitations, the results from our study provide some meaningful suggestions. Design training programs should teach both divergent and convergent thinking to enhance students’ creativity. Both are valued, although the extent to which each type of thinking should be emphasized is an open question. Ideally, research can inform the design training curriculum. Training could include not just learning divergent thinking, but also convergent thinking and cognitive flexibility. Educators could promote divergent thinking and cognitive disinhibition in the early stages of creation, and convergent thinking and cognitive inhibition in the later stages of creation. These three components of creativity could also be used in the assessment of designs in educational settings. Therefore, in future research on creativity, students’ cognitive flexibility, divergent thinking, and convergent thinking are concepts that all need attention.

## Data Availability Statement

The raw data supporting the conclusions of this article will be made available by the authors, without undue reservation.

## Ethics Statement

The studies involving human participants were reviewed and approved by Academic Ethics Committee of Guangdong University of Technology. The patients/participants provided their written informed consent to participate in this study.

## Author Contributions

TX and MC designed the study. MC, MK, and JO collected and analyzed the data. TX, MK, and FH wrote the first draft of the manuscript. TX revised the manuscript. All authors contributed to the article and approved the submitted version.

## Funding

This research was funded by grants from the National Social Science Foundation of China (18BYY089).

## Conflict of Interest

The authors declare that the research was conducted in the absence of any commercial or financial relationships that could be construed as a potential conflict of interest.

## Publisher’s Note

All claims expressed in this article are solely those of the authors and do not necessarily represent those of their affiliated organizations, or those of the publisher, the editors and the reviewers. Any product that may be evaluated in this article, or claim that may be made by its manufacturer, is not guaranteed or endorsed by the publisher.
